# Effects of Oligomeric Proanthocyanidins on Cadmium-Induced Extracellular Matrix Damage via Inhibiting the ERK1/2 Signaling Pathway in Chicken Chondrocytes

**DOI:** 10.3390/vetsci12040317

**Published:** 2025-03-31

**Authors:** Jianhong Gu, Dan Liu, Anqing Gong, Xinrui Zhao, Jiatao Zhou, Panting Wang, Han Xia, Ruilong Song, Yonggang Ma, Hui Zou, Muhammad Azhar Memon, Yan Yuan, Xuezhong Liu, Jianchun Bian, Zongping Liu, Xishuai Tong

**Affiliations:** 1 College of Veterinary Medicine, Joint International Research Laboratory of Agriculture and Agri-Product Safety of the Ministry of Education of China, Yangzhou University, Yangzhou 225009, China; jhgu@yzu.edu.cn (J.G.); dliu08041@163.com (D.L.); mz120221680@stu.yzu.edu.cn (A.G.); 222001134@stu.yzu.edu.cn (X.Z.); 222001135@stu.yzu.edu.cn (J.Z.); mx120221015@stu.yzu.edu.cn (P.W.); mx120221012@stu.yzu.edu.cn (H.X.); rlsong@yzu.edu.cn (R.S.); 007854@yzu.edu.cn (Y.M.); zouhui@yzu.edu.cn (H.Z.); yuanyan@yzu.edu.cn (Y.Y.); xzliu@yzu.edu.cn (X.L.); jcbian@yzu.edu.cn (J.B.); liuzongping@yzu.edu.cn (Z.L.); 2 Jiangsu Co-Innovation Center for Prevention and Control of Important Animal Infectious Diseases and Zoonoses, Yangzhou 225009, China; 3 Jiangsu Key Laboratory of Zoonosis, Yangzhou 225009, China; 4 MOE Joint International Research, College of Veterinary Medicine, Nanjing Agricultural University, Nanjing 210095, China; dr_azharmemon@stu.njau.edu.cn

**Keywords:** oligomeric proanthocyanidins (OPCs), cadmium (Cd), ERK1/2 signaling pathway, chondrocyte, chicken

## Abstract

Cadmium (Cd) is a toxic, non-essential metal that primarily enters animal bodies through the digestive and respiratory systems, leading to damage to multiple organs and tissues. Cd can accumulate in cartilage and induce damage to chondrocytes. Procyanidins (PAs), also known as concentrated tannic acid or oligomeric proanthocyanidins (OPCs), exhibit diverse biological and pharmacological activities. However, the mechanism of OPCs alleviates Cd-induced damage to chondrocytes in chickens remains to be further explored in vitro. Chondrocytes were isolated from both ends of the tibia of 17-day-old SPF chicken embryos, and then subsequently treated with various concentrations of Cd (0, 1, 2.5, 5, and 10 μmol/L) or OPCs (0, 5, 10, 20, and 40 μmol/L) to investigate the mechanism underlying extracellular matrix (ECM) degradation and damage. Cd reduced cell viability, glycosaminoglycan (GAG) secretion, and ECM degradation in chondrocytes by decreasing the expression of type II collagen alpha 1 (COL2A1) and aggrecan (ACAN) while increasing the release of cartilage oligomeric matrix protein (COMP), along with elevated levels of matrix-degrading enzymes, such as matrix metalloproteinases 1 (MMP1), MMP10, and MMP13, and a disintegrin and metalloproteinase with thrombospondin motifs 4 (ADAMTS4) and ADAMTS5. Cd induced phosphorylation of extracellular signal-regulated kinases 1/2 (ERK1/2) and the expression of matrix-degrading enzymes, impairing ECM synthesis, an effect that could be alleviated by ERK1/2 inhibitor U0126. Chondrocytes were treated with 5 μmol/L Cd and 10 μmol/L OPCs, and it was found that OPCs inhibited the activation of the ERK1/2 signaling pathway and the expression of matrix-degrading enzymes, while promoting ECM synthesis and alleviating Cd-induced ECM damage in chickens. This study provides a theoretical basis for clinical research on OPCs with respect to the prevention and treatment of Cd-induced chondrogenic diseases in poultry.

## Simple Summary

Cadmium (Cd) is a highly toxic industrial pollutant and environmental contaminant that can accumulate in cartilage and induce damage to chondrocytes. Cartilage consists of chondrocytes and an extracellular matrix (ECM). Cd-induced chondrocyte damage may be associated with the pathogenesis of osteoarthritis (OA). ECM degradation in joint and cartilage occurs when catabolism exceeds anabolism. The degradation of ECM in cartilage primarily involves two factors: matrix metalloproteinases (MMPs) and a disintegrin and metalloproteinase with thrombospondin motifs (ADAMTS), which possess platelet thrombin-sensitive protein domains. In addition, oligomeric proanthocyanidins (OPCs) exhibit diverse biological and pharmacological activities, such as anti-inflammatory, antioxidant, antibacterial, anticancer, and immunoregulation activities, and also exert a protective effect against various types of heavy metal poisoning. In this study, Cd activates the expression of matrix-degrading enzymes and the ERK1/2 signaling pathway of chondrocytes, leading to the damage of ECM in chickens. In contrast, OPCs inhibit the activation of the ERK1/2 signaling pathway and the expression of matrix-degrading enzymes, while promoting ECM synthesis in chondrocytes. These signs indicate that OPCs can alleviate Cd-induced ECM damage in chickens.

## 1. Introduction

Cadmium (Cd) is a highly toxic industrial pollutant and environmental contaminant, characterized by its silver-white color, metallic luster, and high ductility. Cd commonly occurs in nature as sulfides [1]. Cd can contaminate the environment and jeopardize the health of animals and humans through air, water, soil, and food [2]. Both short- or long-term exposure to Cd can cause damage to the bones, nervous, reproductive, and cardiovascular systems [3,4]. The half-life of Cd in human tissues is 10–30 years during the accumulation phase, particularly in muscles, kidney cortex, and liver [5]. Furthermore, exposure to Cd may contribute to the development of skeletal diseases, including osteoporosis (OP), rheumatoid arthritis (RA), and osteoarthritis (OA) [3]. It was initially suggested that the toxic effect of Cd on bones was indirect, occurring through the binding of Cd to metallothioneins (MTs), with subsequent reabsorption and accumulation in the kidneys. Cd competes with transient receptor potential vanilloid 5 (TRPV5) and TRPV6 for binding calcium (Ca), thereby inhibiting Ca absorption [6]. Prolonged exposure to Cd can lead to glomerular damage and a reduction in the glomerular filtration rate, impairing the reabsorption of Ca and phosphorus (P) by the kidney, which subsequently affects Ca and P metabolism in bones [7,8]. Cd can also directly damage bones by inducing apoptosis in bone-related cells, contributing to OP [9]. It has been reported that Cd concentrations ranging from 10 to 100 nmol/L can damage bones by reducing bone mineral density (BMD) and promoting osteoclastic bone resorption [10]. A previous study demonstrated that continuous exposure to 50 mg/L of cadmium chloride (CdCl_2_) for 24 h reduced BMD (by approximately 10%) and trabecular thickness while increasing the number of osteoclasts in the bone trabeculae of 8-week-old male Sprague Dawley rats [11]. Cd also inhibits the differentiation of bone marrow mesenchymal stem cells (BMSCs) into osteoblasts, impeding collagen growth and secretion, as well as inhibiting alkaline phosphatase (ALP) activity, thereby reducing bone mineralization and formation [12,13].

Cd-induced chondrocyte damage may be associated with the pathogenesis of OA. Lanocha et al. reported that OA patients exhibited elevated Cd concentrations in the tibia, with individuals displaying degenerative changes showing higher Cd concentrations in the hip joint compared to those with knee injuries [14]. Another study assessed the correlation between blood Cd levels in smokers and OA severity, finding that the proportion of moderate and severe OA cases was higher among smokers. Furthermore, Cd in tobacco was associated with more extensive cartilage loss compared to non-smokers [15]. In a three-dimensional chondrocyte culture, exposure to 5 μmol/L CdCl_2_ for 12 h induces inflammatory responses related to interleukin-1 beta (IL-1β) and IL-6, while also promoting the expression of matrix-degrading enzymes in articular chondrocytes. This subsequently affects the expression of type II collagen alpha 1 (COL2A1) and aggrecan (ACAN), ultimately leading to cartilage damage [16]. Cartilage consists of chondrocytes and an extracellular matrix (ECM), which is characterized by a dense collagen fiber network (comprising approximately 60% of its dry weight) and is primarily composed of water, collagen, polysaccharides (PGs), and non-collagen proteins [17,18]. Type II collagen is the predominant collagen protein in cartilage ECM, and the core fiber network is a cross-linked copolymer of type II, VI, and XI collagens, which play a crucial role in cartilage development [19]. Consequently, the collagen fiber network provides tensile strength to the cartilage matrix, while PGs contribute to the osmotic swelling and elastic properties of cartilage tissue [20]. The water in the ECM is not free but is attracted to the matrix by highly negatively charged glycosaminoglycans (GAGs). GAGs are highly hydrophilic macromolecules composed of polysaccharide chains of repeated disaccharide units, capable of binding to the central core protein of proteoglycans [21]. Furthermore, PGs can bind various morphogens and growth factors through the GAG side chain [22]. The PG family of the ECM can be classified into two categories: aggregated and non-aggregated. ACAN is highly expressed in cartilage and is characteristic by its ability to interact with hyaluronic acid (HA) to form large protein polysaccharide aggregates [23,24]. Additionally, cartilage oligomeric matrix protein (COMP), also known as thrombospondin (TSP)-5, is a non-collagenous glycoprotein of the ECM, belonging to the TSP family [25]. COMP interacts with numerous other ECM components and binds to various cell receptors and growth factors, serving as a bridging molecule that regulates cell phenotype and function [26].

In healthy cartilage, the synthesis and catabolism of the ECM are generally balanced. ECM degradation and joint damage in cartilage occur when catabolism exceeds anabolism [27]. The degradation of the ECM in cartilage primarily involves two factors: matrix-degrading enzymes and regulatory factors. Matrix-degrading enzymes primarily include matrix metalloproteinases (MMPs) and a disintegrin and metalloproteinase with thrombospondin motifs (ADAMTS), which possess platelet thrombin-sensitive protein domains. MMPs are a class of zinc (Zn)/Ca-dependent peptide chain proteases capable of degrading and modifying most ECM components [28]. Classic MMPs include MMP1, MMP9, and MMP13, with MMP13 being considered the most significant factor in the degradation of collagen in cartilage, primarily due to its preferential digestion of type II collagen [29]. Excessive MMP13 activity can lead to cartilage degradation and joint pathological changes, suggesting that it plays a key role in the onset of OA. However, intraperitoneal injection of CL82198 (an MMP-13 inhibitor) slowed the progression of OA compared to saline treatment [30]. ADAMTS4 and ADAMTS5 are key members of the ADAMTS metalloproteinase family, involved in the degradation and cleavage of the Glu^373^-Ala^374^ bond in the interglobular domain (IGD) of ACAN [31]. ADAMTS4 is primarily expressed in cartilage affected by OA, whereas ADAMTS5 is expressed in both OA and healthy cartilage tissues. Genetic polymorphism in ADAMTS5 is associated with susceptibility to OA. In an experimental model of cartilage explants from OA, silencing of ADAMTA4 and ADAMTS5 was found to decelerate cartilage degeneration, with a significant increase in ADAMTS4 mRNA expression [32].

Procyanidins (PAs) are naturally occurring polyphenolic compounds found in plants and are abundant in edible plants such as vegetables, fruits, nuts, and spices, and berries, with grapes and cranberries being among the richest sources [33,34]. PAs are compounds formed by the polymerization of flavan-3-ol through C-C bonds and can be classified into monomeric, oligomeric (2–6 monomer), and polymeric (10 or more monomer) forms based on the t degree of polymerization [35]. PAs are safe for consumption and possess anti-inflammatory, antioxidant, antibacterial, anticancer, and immunoregulation properties. Aflatoxin B_1_ (AFB_1_) is a widespread fungal toxin that can contaminate food and feed, leading to severe oxidative stress damage and immune toxicity. However, PAs alleviate AFB_1_-induced immune toxicity and oxidative damage by inhibiting nuclear factor kappa-light-chain-enhancer of activated B cells (NF-κB) and activating the nuclear factor-erythroid-2-related factor (Nrf2) signaling pathway in broiler chickens [36]. Supplementing PAs in the diet can improve the liver function, reduce fat deposition, lower body fat content, decrease cholesterol content in egg yolks and ovarian steroids, enhance antioxidant capacity, and improve egg quality in laying hens [37]. Additionally, supplementing 200 mg/kg of PAs in the diet of piglets significantly increases the thickness and surface area of colon mucosa, regulates intestinal microbiota, and enhances antioxidant capacity and lipid metabolism in the colon [38]. PAs also exert a protective effect against various types of heavy metal poisoning. Chen et al. reported that PAs protect against Cd-induced renal oxidative damage in mice and may inhibit Cd-induced renal cell apoptosis by modulating the expression of B-cell lymphoma 2 (Bcl-2) and Bcl-2 associated X-protein (Bax) [39]. Gong et al. found that PAs (also known as OPCs) alleviate kidney damage by regulating the oxidative-antioxidant balance, including lipid peroxidation, protein carbonyl (PCO), superoxide dismutase (SOD), glutathione S transferase (GST), and glutathione peroxidase (GSH-Px). Additionally, PAs enhance the chelating capacity of Cd, promote its excretion via urine, regulate levels of essential elements (Zn, Ca, Cu, and Fe), and activate the p38 mitogen-activated protein kinase (MAPK) and Keap1/Nrf2 signaling pathways [40]. PAs also inhibit the activation of cell apoptosis and activate the Nrf2 pathway by restoring normal tissue structure and regulating the cytosolic antioxidant enzyme system in the testes, thus providing protection to the testes of Cd-poisoned rats [41,42]. However, the mechanism of action of OPCs in Cd-induced chondrocytes of laying hens has not been reported.

This study investigates chondrocytes derived from chicken embryos cultured in vitro. The study examines the effect of OPCs on Cd-induced damage to the ECM of chondrocytes in chickens, with a focus on the extracellular signal-regulated kinases 1/2 (ERK1/2) signaling pathway and MMPs. The aim is to provide a theoretical basis for clinical research on the use of OPCs in the prevention and treatment of Cd-induced chondrogenic diseases in poultry.

## 2. Materials and Methods

### 2.1. Reagents

Dulbecco modified Eagle’s medium/nutrient mixture (DMEM)/F-12 was purchased from Gibco (Grand Island, NY, USA). Fetal bovine serum (FBS) was purchased from GeminiBio (West Sacramento, CA, USA). CdCl_2_ was obtained from Sigma-Aldrich (St. Louis, MO, USA). Type IV collagenase was sourced from Biosharp (Hefei, Anhui, China). A mixture of protease and phosphatase inhibitors, a radioimmunoprecipitation assay (RIPA) kit, 4% paraformaldehyde solution, 0.25% trypsin solution, and an electrochemiluminescence (ECL) chemiluminescence kit were purchased from Suzhou New Cell & Molecular Biotech Co., Ltd. (Suzhou, Jiangsu, China). TRIzol reagent was purchased from Invitrogen (Carlsbad, CA, USA). HiScript Q RT SuperMix was purchased from Vazyme (Nanjing, Jiangsu, China). The Hieff qPCR SYBR Green Master Mix kit was purchased from Yeasen Biotechnology Co., Ltd. (Shanghai, China). L-glutamine, non-fat milk powder, and an Alcian blue staining kit were obtained from Beijing Solarbio Science & Technology Co., Ltd. (Beijing, China). A cell counting (CCK-8) assay kit and BCA protein assay kit were purchased from Yeasen Biotechnology (Shanghai) Co., Ltd. (Shanghai, China). A standard protein marker was purchased from Laboratories Inc. (Hercules, CA, USA). Antibodies against COL2A1 and aggrecan (ACAN) were purchased from Merck (Rahway, NJ, USA) and Santa Cruz Biotechnology Inc (Santa Cruz, CA, USA), respectively. Antibodies against phosphorylated (p)-ERK1/2, ERK1/2, horseradish peroxidase (HRP)-conjugated goat anti-mouse, and HRP-conjugated goat anti-rabbit were obtained from Cell Signaling Technology, Inc. (Danvers, MA, USA). The Chicken COMP enzyme-linked immunosorbent kit was purchased from Shanghai Enzyme-linked Biotechnology Co., Ltd. (Shanghai, China). U0126 was purchased from Absin Bioscience Inc. (Shanghai, China). OPCs were purchased from Shanghai Yuanye Biotechnology Co., Ltd. (Shanghai, China).

### 2.2. SPF Grade Eggs

SPF-grade White Leghorn eggs were purchased from Zhejiang Lihua Agricultural Technology Co., Ltd. (Ningbo, Zhejiang, China). The fertilized eggs were incubated in an egg incubator (Dezhou Dingfeng Machinery Equipment Co., Ltd.; Dezhou, Shandong, China). All chicken embryos were humanely euthanized, and cartilage tissue from the legs was separated for further testing at day 17 of incubation. The experimental protocol followed was approved by the Animal Care and Use Committee of Yangzhou University (Approval ID: SYXK [Su] 2021–0027) and strictly followed the guidelines set forth by the National Research Council for the Care and Use of Laboratory Animals.

### 2.3. Isolation of Chondrocytes in Chicken Embryo Joints

Cartilage tissue from the joints of SPF-grade chicken embryos was carefully separated, and the surrounding connective tissue was removed. The cartilage tissue was then cut into a small piece (with a size of 1–3 mm) and washed three times with phosphate buffered saline (PBS). The cartilage tissue was digested with 0.2% type IV collagenase overnight. The following day, the supernatant was collected and filtered through a 400-mesh filter. The cell suspension was centrifuged at 1300 rpm/min for 6 min. The cells were resuspended in DMEM/F-12 (containing 10% FBS) after removing the supernatant. Cells were seeded into a dish with a diameter of 60 mm or 100 mm and incubated at 37 °C with 5% CO_2_. The medium was replaced every 48 h.

### 2.4. Identification of Chondrocytes

To further confirm the differentiation of chondrocytes, an Alcian blue staining kit was used to determine the secretion of GAG. The culture medium was discarded, and the cells were washed three times with PBS. Cells were then fixed in 4% paraformaldehyde solution for 10 min at room temperature (25 °C) and washed three times with PBS. Staining was carried out according to the corresponding manufacturer’s instructions, as previously reported [43]. The stained cells were observed under a DMI 3000 B inverted phase contrast microscope (Leica, Germany).

### 2.5. Measurement of Cell Proliferation of Chondrocytes

Cells were seeded into a 96-well plate at a density of 5 × 10^4^ cells/well and cultured until they reached approximately 70% confluence. The medium was replaced every 48 h. A total of 1.83 mg of CdCl_2_ powder was dissolved in 10 mL of ultrapure water to prepare a 1 mmol/L solution (as the Cd group) for later use. Various concentrations of Cd (0, 1, 2.5, 5 and 10 μmol/L) were added to cell cultures for 12 h or 24 h. After the treatment, the supernatant was discarded, and the cells were washed three times with PBS. Cell viability was assessed using a CCK-8 assay kit as previously reported [44]. Furthermore, various concentrations of U0126 (0, 5, 10, and 20 μmol/L) were added to the cell culture medium for pre-treatment for 1 h, followed by the addition of 5 μmol/L Cd to the same medium for 24 h. Alternatively, cells were co-treated with 5 μmol/L Cd and varying concentrations of OPCs (0, 5, 10, 20, and 40 μmol/L) for 24 h. After incubation, the cells were subjected to subsequent experiments.

### 2.6. ELISA Analysis

Cells were seeded into a 24-well plate at a density of 1 × 10^5^ cells/mL and cultured until they reached approximately 80% confluence. The medium was replaced every 48 h. Various concentrations of Cd (0, 1, 2.5, 5 and 10 μmol/L) were added to the cell culture for 24 h. To investigate the changes to COMP, the supernatant was collected into 1.5 mL tubes and centrifuged at 1000 g/min for 20 min. Briefly, standard wells (50 μL standard sample at different concentrations), blank wells (without any reagents), and testing wells (50 μL samples of the supernatant per well) were set up. Then, 100 μL of HRP-labeled antibody was added to each well. The plate was incubated at 37 °C for 60 min. After incubation, the supernatant was discarded and 350 μL of washing liquid was added to each well, followed by a 1 min incubation. This washing step was repeated five times. Then, 50 μL of the substrate A and B were added to each well, and the plate was incubated at 37 °C for 15 min, protecting it from light. The blank wells were set to zero, and the absorbance of the other wells was determined as previously reported [45]. The absorbance of each well was measured using a BioTek SynergyHTX Multimode Reader (Winooski, VT, USA). The concentration of COMP was measured according to the corresponding manufacturer’s instructions.

### 2.7. Quantitative Real-Time Polymerase Chain Reaction (qRT-PCR)

Cells were seeded into a dish with a diameter of 60 mm at a density of 1 × 10^5^ cells/mL and cultured until they reached approximately 80% confluence. The medium was replaced every 48 h. Various concentrations of Cd (0, 1, 2.5, 5 and 10 μmol/L) were added to the cell for 24 h. The supernatant was removed, and total RNA was extracted from the cells using TRIzol reagent as previously reported [46]. Pure total RNA was then reverse-transcribed into complementary DNA (cDNA) using the HiScript Q RT SuperMix kit according to the corresponding manufacturer’s instructions. Primer sequences (Table 1) were used to perform the qRT-PCR. The cDNA template was used to detect the relative mRNA expression of target genes using the Hieff qPCR SYBR Green Master Mix kit according to the corresponding manufacturer’s instructions. The PCR conditions were as follows: pre-denaturation at 95 °C for 30 s, followed by 40 cycles at 95 °C for 5 s and 60 °C for 34 s. The relative expression of target genes was calculated using the 2^−ΔΔCT^ method. Glyceraldehyde-3-phosphate dehydrogenase (GAPDH) was used as the internal control for normalization.

### 2.8. Western Blotting

Cell samples were collected for total protein extraction using the RIPA assay kit, which contains a mixture of protease and phosphatase inhibitors, and incubated on ice for 30 min. Protein concentrations were determined using the BCA protein assay kit as previously described [46]. Equal amounts of total protein (30 μg) were separated by 8–12% sodium dodecyl sulfate-polyacrylamide gel electrophoresis (SDS-PAGE) gels. The proteins were transferred to polyvinylidene fluoride (PVDF) membranes (0.22 μm) after electrophoresis. The membranes were incubated with 5% non-fat milk dissolved in tris-buffered saline solution with 0.1% Tween-20 (TBST) buffer at room temperature (25 °C) for 2 h. Primary antibodies against COL2A1, ACAN, ERK1/2, p-ERK1/2, and GAPDH were incubated with the membranes at 4 °C overnight. After washing with TBST buffer, the membranes were incubated with the HRP-conjugated goat anti-mouse and HRP-conjugated goat anti-rabbit secondary antibodies at room temperature (25 °C) for 2 h. Finally, the blots were visualized using an ECL chemiluminescence kit and a Tanon-5200 ECL chemiluminescent imaging system (Shanghai, China). The band intensities were quantitatively analyzed for statistical calculation.

### 2.9. Statistical Analysis

Statistical analysis was performed using one-way analysis of variance (ANOVA) with SPSS 25.0 (IBM SPSS Inc.; Chicago, IL, USA). Intergroup differences were analyzed through multiple comparisons. *p* < 0.05 and *p* < 0.01 were considered statistically significant and highly significant, respectively. Data are presented as means ± standard deviation (SD). All experiments were conducted in triplicate.

## 3. Results

### 3.1. Effects of Cd on the Viability of Chondrocytes in Chickens In Vitro

**Figure 1:**
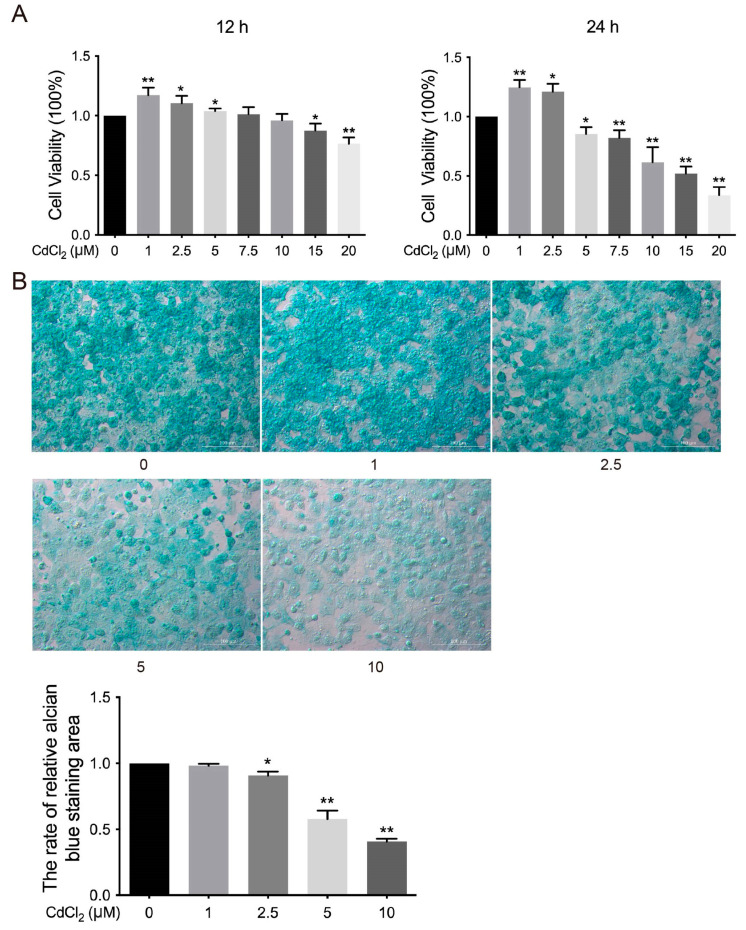
Effects of Cd on the viability of chondrocytes in chickens in vitro. (**A**) Cell viability of chondrocytes was assessed by CCK-8 assay kit. (**B**) GAG secretion of chondrocytes was observed. * *p* < 0.05 or ** *p* < 0.01 vs. 0 μmol/L Cd group.

To investigate the effect of Cd on the viability of chicken chondrocytes in vitro, cell viability was assessed after treatment with different concentrations of Cd (0, 1, 2.5, 5, 7.5, 10, 15, and 20 μmol/L) for 12 h or 24 h. The results indicated that cell viability was significantly increased (* *p* < 0.05 and ** *p* < 0.01) in the 1, 2.5, and 5 μmol/L Cd-treated groups compared to the 0 μmol/L Cd group after 12 h of treatment. In contrast, cell viability was significantly decreased (* *p* < 0.05 and ** *p*< 0.01) in the 15 and 20 μmol/L Cd-treated group (Figure 1A). Similarly, the 1 and 2.5 μmol/L Cd-treated group showed significant (* *p* < 0.05 and ** *p* < 0.01) increases in cell viability compared to the 0 μmol/L Cd group. However, the 5, 7.5, 10, 15, and 20 μmol/L Cd-treated groups exhibited significant (* *p* < 0.05 and ** *p* < 0.01) inhibitory effects on cell viability (Figure 1A). Furthermore, the expression of GAG in chondrocytes was significantly decreased (* *p* < 0.05 and ** *p* < 0.01) in the 2.5, 5, and 10 μmol/L Cd-treated groups compared to the 0 μmol/L Cd group (Figure 1B). These findings suggest that Cd inhibits both cell viability and GAG expression in chicken chondrocytes in vitro.

### 3.2. Effects of Cd on the ECM of Chondrocytes in Chickens In Vitro

**Figure 2:**
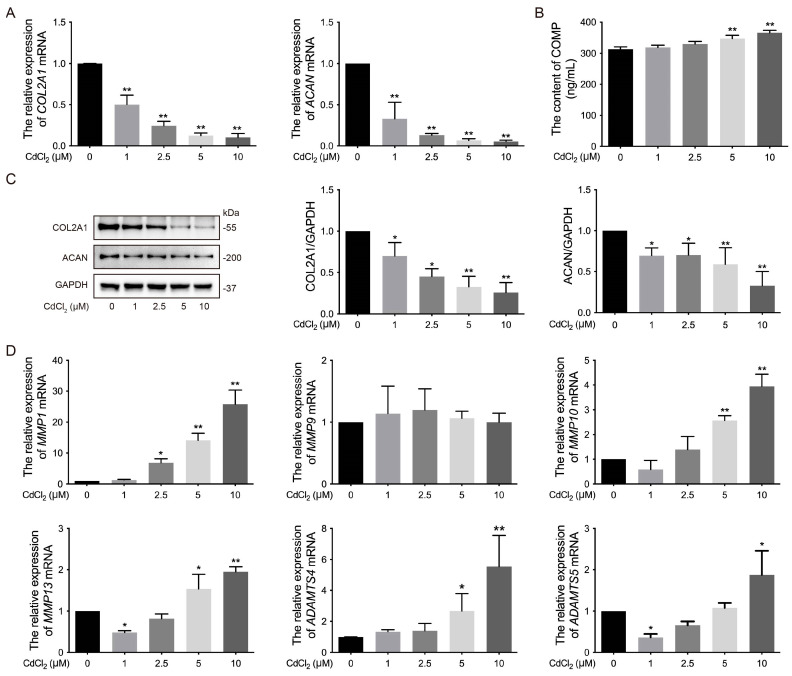
Effects of Cd on the ECM of chondrocytes in chickens in vitro. (**A**) The mRNA levels of *COL2A1* and *ACAN* were measured by qRT-PCR. (**B**) The level of COMP in cultural supernatant was determined using an ELISA kit. (**C**) The expression of COL2A1 and ACAN was quantified by Western blot. (**D**) The mRNA levels of *MMP1*, *MMP9*, *MMP10*, *MMP13*, *ADAMTS4*, and *ADAMTS5* were analyzed by qRT-PCR. * *p* < 0.05 or ** *p* < 0.01 vs. 0 μmol/L Cd group.

The ECM exhibits special fluid pressurization characteristics and long-term load-bearing capacity [47]. Notably, the ECM is composed of type II collagen and ACAN [48,49]. To assess the impact of Cd on the key regulatory factors of chondrocytes in vitro, the expression of ECM-related markers was analyzed after treatment with various concentrations of Cd. The results showed that the mRNA levels of *COL2A1* and *ACAN*, as well as the protein expression of COL2A1 and ACAN, were significantly (* *p* < 0.05 and ** *p* < 0.01) lower in the 1, 2.5, 5, and 10 μmol/L Cd-treated groups compared to the 0 μmol/L Cd group (Figure 2A,C, The original images are attached in Supplementary Materials). Additionally, COMP is a glycoprotein in the ECM that is crucial for the stability of ECM and collagen assembly [26]. The content of COMP in the supernatant of the chondrocyte culture medium was significantly (** *p* < 0.01) increased in the 5 and 10 μmol/L Cd-treated groups compared to the 0 μmol/L Cd group (Figure 2B). Furthermore, secreted COMP is involved in ECM component interactions that influence cell signaling and chondrocyte function, including the regulation of MMPs, such as MMP1, MMP3, MMP10, MMP13, ADAMTS4, and ADAMTS5 [50]. The mRNA levels of *MMP1*, *MMP3*, *MMP10*, *MMP13*, *ADAMTS4*, and *ADAMTS5* were significantly (* *p* < 0.05 and ** *p* < 0.01) higher in the Cd-treated groups compared to the 0 μmol/L Cd group, except for *MMP9*, which showed no significant difference (Figure 2D). These results demonstrate that Cd promotes ECM degradation by increasing the expression of matrix-degrading enzymes and inhibiting the synthesis of ECM components in chicken chondrocytes in vitro.

### 3.3. Effects of the ERK Signaling Pathway on Cd-Induced Phosphorylation of ERK1/2 of Chondrocytes in Chickens In Vitro

**Figure 3:**
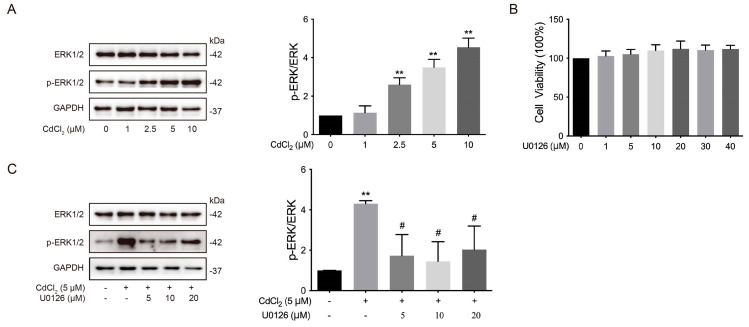
Effects of ERK signaling pathway on Cd-induced phosphorylation of ERK1/2 in chicken chondrocytes in vitro. (**A**) The phosphorylation of ERK1/2 was analyzed by Western blot. ** *p* < 0.01 vs. 0 μmol/L Cd group. (**B**) Cell viability of chondrocytes was assessed with different concentrations of U0126 using a CCK-8 assay kit. (**C**) The phosphorylation of ERK1/2 was measured by Western blot. ** *p* < 0.01 vs. 0 μmol/L Cd, # *p* < 0.05 vs. 5 μmol/L Cd group.

The ERK signaling pathway is a major branch of the MAPK pathway, playing a crucial role in cell proliferation, apoptosis, DNA repair, and cell metabolism. Importantly, Cd has been shown to activate the ERK1/2 signaling pathway in osteoblasts and neuronal cells [51,52]. As shown in the Figure 3A (The original images are attached in Supplementary Materials), the phosphorylation of ERK1/2 was significantly (** *p* < 0.01) higher in the 2.5, 5, and 10 μmol/L Cd-treated groups compared to the 0 μmol/L Cd group. Furthermore, the ERK1/2 inhibitor U0126 had no significant effects on the cell viability of chicken chondrocytes (Figure 3B). However, the phosphorylation of ERK1/2 in the Cd + U0126 co-treated group was significantly (# *p* < 0.05) lower than in the Cd-treated group (Figure 3C, The original images are attached in Supplementary Materials). These findings suggest that U0126 reduces Cd-induced ERK1/2 phosphorylation in chicken chondrocytes via the ERK1/2 signaling pathway in vitro.

### 3.4. Effects of the ERK Signaling Pathway on Cd-Induced ECM Damage in Chicken Chondrocytes In Vitro

**Figure 4:**
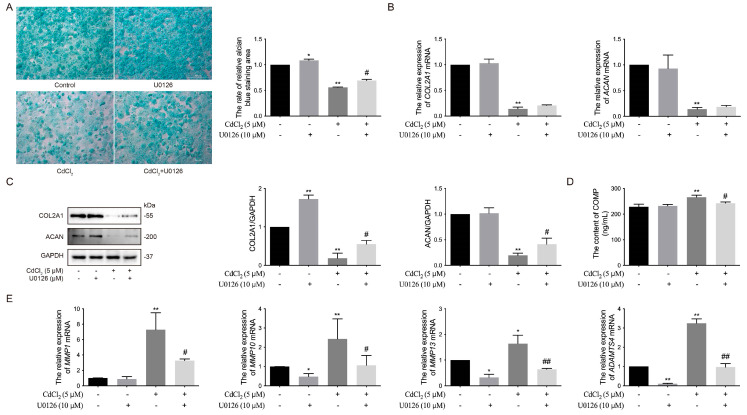
Effects of the ERK signaling pathway on Cd-induced ECM damage to chondrocytes in chickens in vitro. (**A**) GAG secretion of chondrocytes was observed. (**B**) The mRNA levels of *COL2A1* and *ACAN* were measured by qRT-PCR. (**C**) The expression of COL2A1 and ACAN was quantified by Western blot. (**D**) The level of COMP in the cultural supernatant was detected by ELISA. (**E**) The mRNA levels of *MMP1*, *MMP10*, *MMP13,* and *ADAMTS4* were analyzed by qRT-PCR. * *p* < 0.05 or ** *p* < 0.01 vs. control (only cells), # *p* < 0.05 or ## *p* < 0.01 vs. 5 μmol/L Cd group.

To further investigate the impact of the ERK1/2 signaling pathway on the expression of GAGs in chondrocytes treated with Cd and the ERK1/2 inhibitor U0126, the results revealed that the expression of GAGs in the 5 μmol/L Cd group was significantly (** *p* < 0.01) lower than in the control group, whereas the 10 μmol/L U0126 group exhibited significantly (* *p* < 0.05) higher GAG expression compared to the control group (Figure 4A). Additionally, the expression of GAGs in the Cd + U0126 co-treated group was significantly (# *p* < 0.05) higher than in the Cd group (Figure 4A). Similarly, the mRNA levels of *COL2A1* and *ACAN* in the 5 μmol/L Cd group were significantly (** *p* < 0.01) lower than in the control group, whereas the 10 μmol/L U0126 co-treated group, with or without Cd, showed no significant differences compared to the control group (Figure 4B). Correspondingly, the protein expression of COL2A1 and ACAN in the 5 μmol/L Cd group was significantly (** *p* < 0.01) lower than in the control group, while the 10 μmol/L U0126 group exhibited a significant (** *p* < 0.01) increase in COL2A1 expression (Figure 4C, The original images are attached in Supplementary Materials). Likewise, the expression of COL2A1 and ACAN in the Cd + U0126 co-treated group was significantly (# *p* < 0.05) higher than in the Cd group (Figure 4C, The original images are attached in Supplementary Materials). The content of COMP in the supernatant of chondrocyte culture medium was significantly (** *p* < 0.01) higher in the 5 μmol/L Cd group compared to the control group but was significantly (# *p* < 0.05) lower in the Cd + U0126 co-treated group compared to the Cd group (Figure 4D). Moreover, the mRNA levels of *MMP10*, *MMP13*, and *ADAMTS4* were significantly (* *p* < 0.05 and ** *p* < 0.01) lower in the Cd-treated groups compared to the control group, except for *MMP1*, which showed no significant difference. These mRNA levels were further reduced by U0126 treatment (Figure 4E). These results suggest that Cd promotes the expression of matrix-degrading enzymes and inhibits ECM synthesis components in chondrocytes by activating the ERK1/2 signaling pathway, leading to ECM damage and cartilage degradation in chickens in vitro.

### 3.5. Effects of OPCs on the Cd-Induced ERK Signaling Pathway of Chondrocytes in Chickens In Vitro

**Figure 5:**
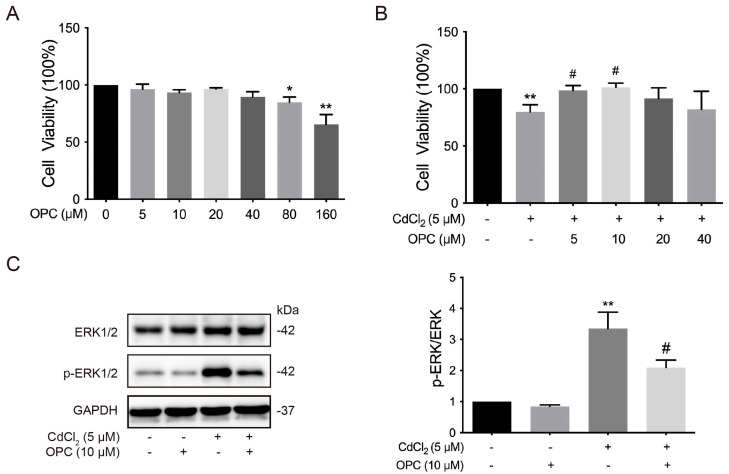
Effects of OPCs on Cd-induced ERK signaling pathway activation in chondrocytes in chickens in vitro. (**A**,**B**) Cell viability of chondrocytes was assessed using the CCK-8 assay kit. (**C**) The phosphorylation of ERK1/2 was measured by Western blot. * *p* < 0.05 or ** *p* < 0.01 vs. control (only cells), # *p* < 0.05 vs. 5 μmol/L Cd group.

OPCs are polyphenolic flavonoid compounds known for their various biological effects, including promoting cell proliferation, scavenging free radicals, and exhibiting antioxidative properties [53]. To examine the role of OPCs in the ERK signaling pathway in chicken chondrocytes, we first evaluated the cell viability of chondrocytes. The results showed that cell viability was significantly (* *p* < 0.05 and ** *p* < 0.01) reduced in 80 and 160 μmol/L OPC-treated groups compared to the 0 μmol/L OPC group (Figure 5A). In contrast, cell viability was significantly (# *p* < 0.05) increased in the 5 and 10 μmol/L OPC co-treated with 5 μmol/L Cd groups compared to the 5 μmol/L Cd-only group (Figure 5B). Additionally, the phosphorylation of ERK1/2 was significantly (# *p* < 0.05) reduced in the 10 μmol/L OPC co-treated with 5 μmol/L Cd group compared to the 5 μmol/L Cd-only group (Figure 5C, The original images are attached in Supplementary Materials). These results suggest that OPCs alleviate Cd-induced damage to chondrocytes and reduce the activation of the ERK1/2 signaling pathway in chicken chondrocytes in vitro.

### 3.6. Effects of OPCs on Cd-Induced ECM Damage to Chondrocytes in Chickens In Vitro

**Figure 6:**
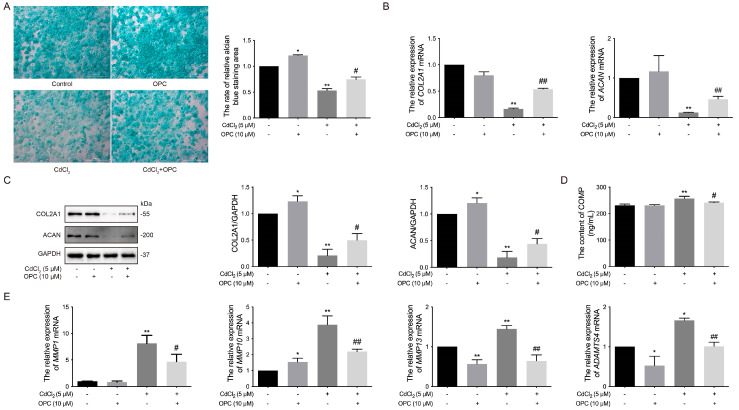
Effects of OPCs on Cd-induced ECM damage to chondrocytes in chickens in vitro. (**A**) GAG secretion by chondrocytes was observed. (**B**) The mRNA levels of *COL2A1* and *ACAN* were measured by qRT-PCR. (**C**) The expression of COL2A1 and ACAN was quantified by Western blot. (**D**) The level of COMP in the cultural supernatant was determined by ELISA. (**E**) The mRNA levels of *MMP1*, *MMP10*, *MMP13*, and *ADAMTS4* were analyzed by qRT-PCR. * *p* < 0.05 or ** *p* < 0.01 vs. control (only cells), # *p* < 0.05 or ## *p* < 0.01 vs. 5 μmol/L Cd group.

OPCs have been shown to prevent cartilage matrix degradation in human cartilage [54]. Cartilage and bone remodeling require the involvement of MMPs, which regulate joint cartilage degeneration and bone remodeling [55,56]. To further investigate the protective effects of OPCs on Cd-induced ECM damage in chicken chondrocytes, we measured the expression of several ECM components and matrix-degrading enzymes. The results revealed that the expression of GAGs, the mRNA levels of *COL2A1* and *ACAN*, and the protein expression of COL2A1 and ACAN were all significantly (# *p* < 0.05 or # *p* < 0.01) higher in the 10 μmol/L OPC co-treated with 5 μmol/L Cd group compared to the 5 μmol/L Cd-only group (Figure 6A–C, The original images are attached in Supplementary Materials). Moreover, the content of COMP and the mRNA levels of *MMP10*, *MMP1*, *MMP13*, and *ADAMTS4* were significantly (# *p* < 0.05 and # *p* < 0.01) lower in the 10 μmol/L OPC co-treated with 5 μmol/L Cd group compared to the 5 μmol/L Cd group (Figure 6D,E). These findings suggest that OPCs mitigate Cd-induced ECM damage in chondrocytes by inhibiting the ERK signaling pathway and the expression of matrix-degrading enzymes, thereby protecting cartilage structure in chickens in vitro.

## 4. Discussion

Cd is a non-essential heavy metal that can accumulate in the body of both humans and animals through various routes, including the digestive tract, respiratory tract, skin, and others [2]. While the majority of research on Cd-induced bone diseases focuses on OP, studies have examined the effects of Cd on the development and progression of OA. A previous study has shown that chondrocytes are more sensitive to Cd, and concentrations of 5 μmol/L CdCl_2_ can lead to a decrease in chondrocyte activity [57]. The results from this study demonstrated that exposure to 5 μmol/L Cd or higher significantly inhibited chondrocyte viability after 24 h of treatment, which is consistent with an earlier study [57]. The ECM is primarily composed of collagen and proteoglycans, with fibrin and elastin present in lower abundance. COL2A1 is a major structural protein in cartilage and is critical for the formation of heterotypic fibrils, along with type VI, IX, and XI collagens [47]. Non-collagen components, such as ACAN and other proteoglycans, interact with the collagen network to provide compressive strength [58]. ACAN is highly expressed in cartilage and plays an essential role in maintaining its structural integrity. Alterations in the quantity and quality of cartilage matrix components, such as COL2A1 and ACAN, directly contribute to the loss of normal biomechanical properties in bone and joint cartilage [59]. COMP, which binds with type II collagen fibers, stabilizes the collagen network within articular cartilage. A reduction in COMP levels in the ECM leads to the disintegration of growth plates, and the release of COMP during joint erosion may hinder joint repair. Serum COMP levels have been suggested as a prognostic maker for the progression of joint destruction and cartilage turnover [60]. In a study by Zamudio et al., an in vitro 3D cartilage model was used to investigate Cd toxicity, and it was found that Cd damages the ECM by increasing the expression of degradation enzymes and pro-inflammatory cytokines, ultimately affecting the expression of COL2A1, ACAN, PGs, and GAGs, thereby promoting the development of OA [16]. Consistent with these findings, the present study demonstrates that Cd exposure reduces GAG expression and increases COMP release in chicken chondrocytes, indicating that Cd disrupts ECM integrity and accelerates the release of COMP into the cell supernatant.

Matrix-degrading enzymes play a critical role in the regulation of cartilage ECM damage. Enzymes such as MMP1, MMP3, MMP9, MMP10, and MMP13 are closely associated with cartilage injury. MMP1, the most abundant member of the MMP family, plays a significant role in the degradation of type II collagen in cartilage. MMP3 and MMP10, also known as stromelysins-1 and stromelysins-2, respectively, can cleave type II collagen and aggrecan [61]. MMP13, a collagenase, is responsible for degrading the major type II collagen in articular cartilage [62]. MMP9 cleaves ECM components, as well as cytokines and chemokines, thereby enhancing their activity [63]. ADAMTS4 and ADAMTS5 are enzymes involved in the cleavage of aggrecan in the ECM. Inada et al. reported that the hydrolysis of COL2A1 leads to chondrocyte hypertrophy and induces the expression of MMP13 [64]. In the present study, we found that the expression levels of MMP13 and ADAMTS5 were significantly decreased in the 1 μmol/L Cd group, while MMP9 expression showed no significant change. However, in the 5 μmol/L Cd group and higher concentrations, the expression levels of MMP1, MMP10, MMP13, and ADAMTS4 were significantly elevated. These results suggest that Cd exposure at concentrations of 5 μmol/L or higher can inhibit the expression of COL2A1 and ACAN, promote ECM degradation, and increase the expression of matrix-degrading enzymes, thereby damaging the ECM and accelerating cartilage destruction in chicken chondrocytes.

The ERK1/2 is a downstream molecule in the MAPK signaling pathway, playing a crucial role in the production of inflammatory cytokines and the activation of catabolic reactions in chondrocytes. Inhibition of the ERK1/2 signaling pathway has been shown to alleviate the reduction in COL2A1 and ACAN expression without inducing apoptosis in primary rat chondrocytes [65]. Similarly, suppression of MAPK/ERK activity enhances chondrogenesis derived from mesenchymal stem cells in White Leghorn chicken embryos [66,67]. In contrast, overactivation of the ERK1/2 pathway has been linked to numerous diseases, including cancer, inflammation, developmental disorders, and neurological disorders [68]. Dysregulation of the ERK1/2 signaling pathway promotes chondrocyte degeneration and contributes to the development of OA [69]. For instance, Khan et al. demonstrated that ERK1/2 was significantly upregulated in cartilage tissues of severe OA patients treated with Wogonin [70]. Additionally, Chow et al. found that the release of early-stage inflammatory cytokines (e.g., IL-1β) activates the NF-κB, phosphoinositide-3 kinase/protein kinase B (PI3K/AKT), and MAPK/ERK signaling pathways, causing damage to articular cartilage [71]. Consistent with these findings, our study showed that Cd stimulates the phosphorylation of ERK1/2 of chicken chondrocytes.

The ERK1/2 inhibitor U0126 is a specific inhibitor of the ERK1/2 signaling pathway that effectively suppresses ERK1/2 phosphorylation. Zhou et al. reported that U0126 reversed the adverse effects of adipocyte cytokines derived from infrapatellar fat pad (IPFP) of end-stage OA patients on chondrocytes [72]. Furthermore, inhibition of ERK1/2, c-Jun N-terminal kinase (JNK), and p38 MAPK by phellodendron amurense alleviated cartilage and chondrocyte damage in human OA cartilage [73]. Similarly, Zhou et al. found that kinsenoside suppressed the NF-κB signaling pathway and the phosphorylation of ERK1/2, JNK, and p38, reducing subchondral bone destruction and articular cartilage damage in anterior cruciate ligament transection (ACLT) mice [74]. Inhibition of p38 MAPK, ERK1/2, and Src family kinases reduced proteolytic cartilage degradation by blocking MMP synthesis and activity, with ERK1/2 playing an essential role in aggrecanase-mediated aggrecan degradation [75]. Co-culture studies have shown that OA subchondral bone osteoblasts significantly increased MMP2, MMP3, MMP9, ADAMTS4, and ADAMTS5 expression in normal chondrocytes, while OA chondrocytes co-cultured with normal osteoblasts led to elevated MMP1 and MMP2 expression. The addition of the MAPK-ERK inhibitor PD98059 reversed the overexpression of ADAMTS and MMPs under these conditions [76]. In contrast, chemokine CCL2 activated ERK1/2 and p38 via CCR2 treatment in both healthy and OA human chondrocytes, inducing the up-regulation of MMP1, MMP3, MMP13, and tissue inhibitor of metalloproteinases1 (TIMP1) expression in healthy chondrocytes, and MMP1 and MMP3 expression in OA chondrocytes, expect for MMP1 and TIMP1 expression [77]. Our study further revealed that the inhibition of the ERK1/2 signaling pathway increased GAG levels and COL2A1 protein expression. In the Cd + U0126 group, Cd treatment significantly decreased the levels of GAGs, COL2A1, and ACAN, along with reducing the expression of MMP1, MMP3, MMP13, and ADAMTS4 compared to those in the Cd-only group. These findings indicate that ERK1/2 signaling contributes to ECM construction and cartilage integrity.

OPCs, concentrated tannic acid with diverse pharmacological properties, are derived from various plants, including flowers, nuts, fruits, bark, and seeds [53,54]. Toxicological studies on OPCs are limited to evaluations of genetoxicity, short-term repeated-dose toxicity, and carcinogenicity [78]. A subchronic in vivo study showed that the dietary intake of 2.5% grape skin extract or grape seed extract resulted in a no observed adverse effect level (NOAEL) in female rats [79]. In this study, OPC concentration below 40 μmol/L had no effect on chondrocyte viability, whereas a concentration exceeding 80 μmol/L inhibited chondrocyte viability, indicating potential chondrotoxic effects at high concentrations. Previous studies have confirmed that OPCs prevent cartilage matrix degradation by stimulating insulin-like growth factor 1 (IGF1) production in human cartilage [80]. OPCs inhibit excessive MMP activation by regulating their expression and activity, thereby protecting cartilage and bones from degradation and promoting joint stability and repair [55,56]. Consistent with these findings, our data demonstrated that 10 μmol/L OPCs co-treated with 5 μmol/L Cd improved chicken chondrocyte viability in vitro. OPCs have also been reported to promote BMSC proliferation by alleviating oxidative stress and activating the Wnt/β-catenin signaling pathway, thereby enhancing osteoblast differentiation in vitro and accelerating osteogenesis in vivo [81]. Oral administration of OPCs improved collagen synthesis and reduced OA symptoms in mouse cartilage [82]. Similarly, OPCs protect cartilage by inhibiting synovitis, subchondral fractures, and cartilage damage, while reducing reactive oxygen species (ROS) production and MMP13 expression in monosodium iodoacetate (MIA)-induced arthritis in rats [83]. Furthermore, OPCs inhibited lipopolysaccharide (LPS)-induced inflammation, ERK1/2 phosphorylation, ROS generation, and mitochondrial membrane potential depletion in RAW264.7 cells, thereby mitigating cellular damage [84]. OPCs derived from cranberries inhibited collagen and gelatin degradation by recombinant MMP1 and MMP9 and the catalytic activity of *Porphyromonas gingivalis* proteases [85]. Additionally, OPCs reduced the gelatin degradation activity of MMP2 and MMP9 in synovial fluid from OA patients [80]. In this study, OPCs alleviated Cd-induced ECM degradation in chicken chondrocytes by inhibiting cartilage matrix-degrading enzymes and suppressing ERK1/2 phosphorylation.

## 5. Conclusions

The results of this study indicate that Cd activates the expression of matrix-degrading enzymes and the ERK1/2 signaling pathway of chondrocytes, leading to ECM damage in chickens. Furthermore, OPCs inhibit the activation of the ERK1/2 signaling pathway and the expression of matrix-degrading enzymes, while promoting ECM synthesis in chondrocytes. OPCs also alleviate Cd-induced ECM damage in chickens. These findings provide a theoretical basis for the prevention and treatment of Cd-induced chondrogenic diseases in poultry, suggesting the potential clinical applications of OPCs in terms of protecting cartilage and improving joint health.

## Figures and Tables

**Table 1:** The primer sequences of target genes for qRT-PCR.

Gene Name	Primer Sequence (5′–3′)	Gene Number	Length (bp)
*COL2A1*	F: GGAGTTTGGCGTGGATATTGG	NM_204426.2	164
	R: GGGTGGGACTGGTTTGCTTT		
*ACAN*	F: TGGGCGTGCGGACCGTTTA	NM_001396161.1	255
	R: TGGGCTCCAGGGTAGCGATG		
*MMP1*	F: ATTTGATGCCATTACCACTT	XM_040658536.2	172
	R: ACTTCATCCCTTTCAATGTTCT		
*MMP9*	F: GTGCCGTGATAGATGATGCCTTCC	NM_204667.2	95
	R: GTCTGCCTCGCCGCTGTAAATC		
*MMP10*	F: ATCAGGCTCTACAGTGGTG	NM_001278089.2	275
	R: ATGGGATACATCAAGGCAC		
*MMP13*	F: CCCAACCCAAAACATCCCAAAACG	NM_001293090.2	94
	R: TGAAGACCAGCATTTCTCCACGAAG		
*ADAMTS4*	F: GACGGCGTGGGAGAAACAGAAAG	XM_040690827.2	113
	R: GGAGGGGCTGAGGTAGACACAG		
*ADAMTS5*	F: AGAGCAGTGTGAAGCAAGGAATGG	XM_040658789.2	96
	R: CCAGGAAGCACTCCAGCATACTTG		
*GAPDH*	F: ATGGCATCCAAGGAGTGA	NM_204305.2	141
	R: GGGAGACAGAAGGGAACAG		

Note: F represents the forward sequence; R represents the reverse sequence.

## Data Availability

The datasets used and/or analyzed during the current study are available from the corresponding author on reasonable request.

## Supplementary Materials

The following supporting information can be downloaded at: https://www.mdpi.com/article/10.3390/vetsci12040317/s1, Supplementary Materials: Western blot original images.

## Abbreviations

The following abbreviations are used in this manuscript:

Abbreviation	Full name
ACAN	aggrecan
ACLT	anterior cruciate ligament transection
ADAMTS	a disintegrin and metalloproteinase with thrombospondin motifs
AFB_1_	Aflatoxin B_1_
ALP	alkaline phosphatase
BMD	bone mineral density
Bax	Bcl-2 associated X-protein
Bcl-2	B-cell lymphoma 2
BMSCs	bone marrow mesenchymal stem cells
Ca	calcium
CCK-8	cell counting kit-8
Cd	cadmium
CdCl_2_	cadmium chloride
COL2A1	type II collagen alpha 1
COMP	cartilage oligomeric matrix protein
DMEM	dulbecco modified Eagle’s medium/nutrient mixture
ECL	electrochemiluminescence
ECM	extracellular matrix
ERK1/2	extracellular signal-regulated kinases 1/2
FBS	fetal bovine serum
GAGs	glycosaminoglycans
GAPDH	glyceraldehyde-3-phosphate dehydrogenase
GSH-Px	glutathione peroxidase
GST	glutathione S transferase
HA	hyaluronic acid
HRP	horseradish peroxidase
IGD	interglobular domain
IGF1	insulin-like growth factor 1
IPFP	infrapatellar fat pad
JNK	c-Jun N-terminal kinase
LPS	lipopolysaccharide
MAPK	mitogen-activated protein kinase
MIA	monosodium iodoacetate
MMPs	matrix metalloproteinases
MMP1	matrix metalloproteinases 1
MTs	metallothioneins
NOAEL	no observed adverse effect level
NF-κB	nuclear factor kappa-light-chain-enhancer of activated B cells
Nrf2	nuclear factor-erythroid-2-related factor
OA	osteoarthritis
OP	osteoporosis
OPC	oligomeric proanthocyanidins
P	phosphorus
PA	procyanidins
PBS	phosphate buffered saline
PCO	protein carbonyl
PGs	polysaccharides
PI3K/AKT	phosphoinositide-3 kinase/protein kinase B
PVDF	polyvinylidene fluoride
RA	rheumatoid arthritis
RIPA	radioimmunoprecipitation assay
ROS	reactive oxygen species
SD	standard deviation
SDS-PAGE	sodium dodecyl sulfate-polyacrylamide gel electrophoresis
SOD	superoxide dismutase
TBST	tris-buffered saline solution with 0.1% Tween-20
TIMP1	tissue inhibitor of metalloproteinases1
TRPV5	transient receptor potential vanilloid 5
TSP	thrombospondin
Zn	zinc
