# Supplementary material for: Effects of Oligomeric Proanthocyanidins on Cadmium-Induced Extracellular Matrix Damage via Inhibiting the ERK1/2 Signaling Pathway in Chicken Chondrocytes
Source: Vet Sci. 2025 Mar 31;12(4):317. doi: 10.3390/vetsci12040317 (PMC12031251; doi:10.3390/vetsci12040317)
Supplement: Supplementary file 1 [file vetsci-12-00317-s001.zip › Supplementary materials.pdf]

Figure 1

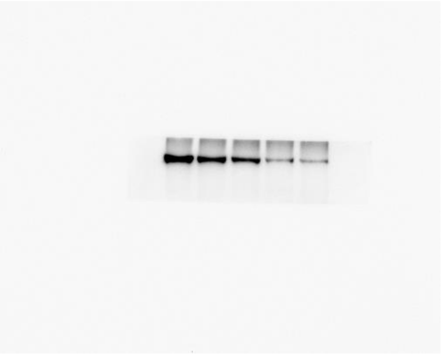

COL2A1

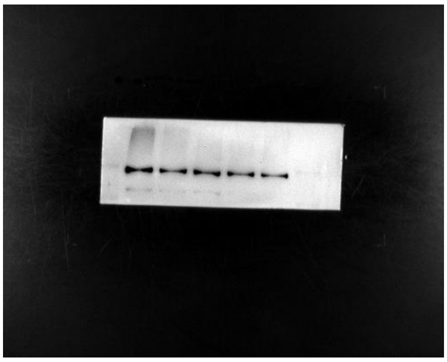

ACAN

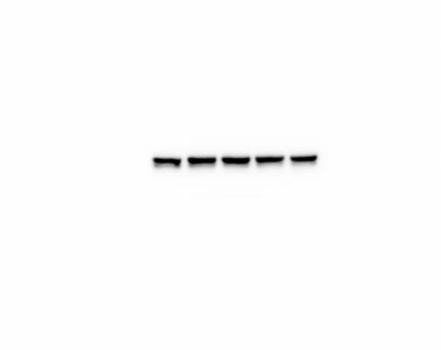

GAPDH

Figure 2

A

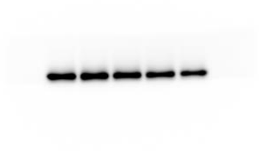

ERK1/2

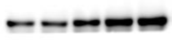

p- ERK1/2

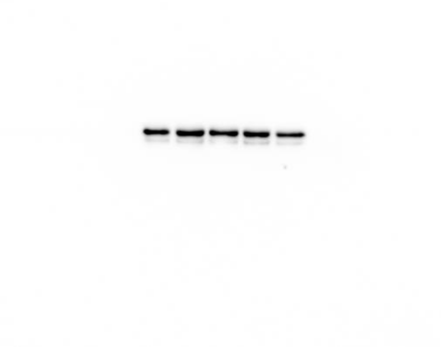

GAPDH

C

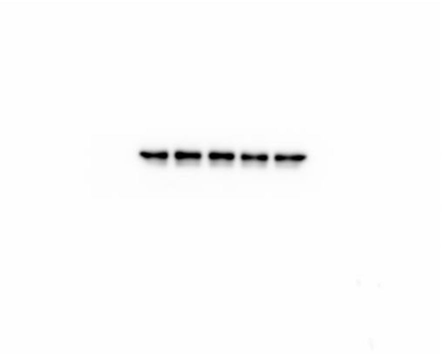

ERK1/2

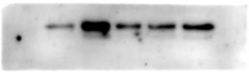

p-ERK1/2

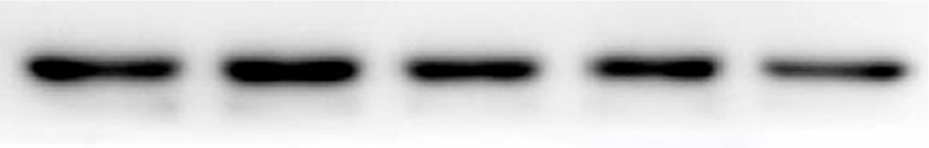

GAPDH

Figure 4

A

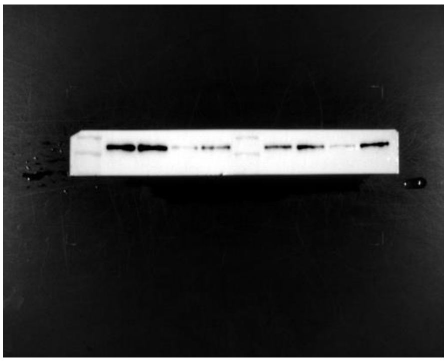

COL2A1 (left)

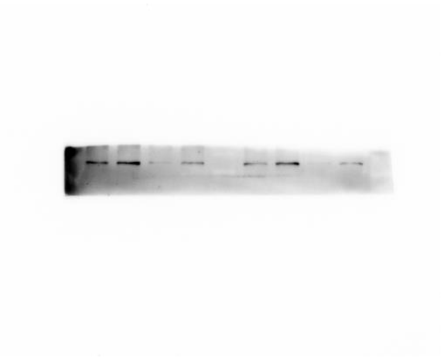

ACAN (left)

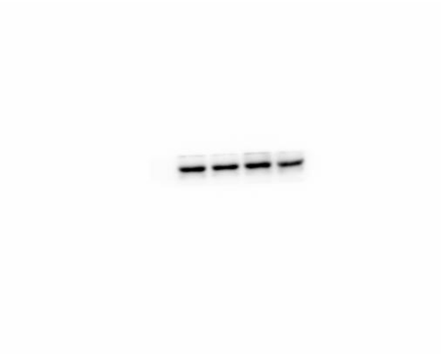

GAPDH

Figure 5

A

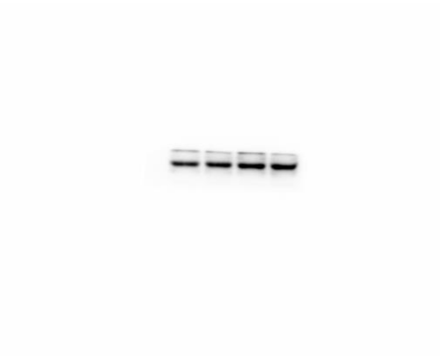

ERK1/2

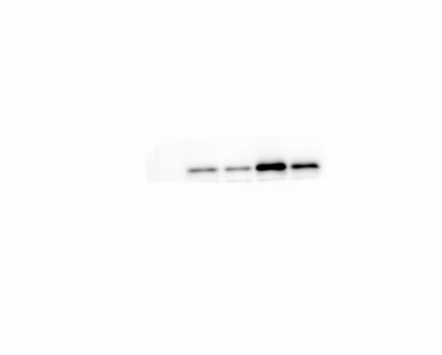

p-ERK1/2

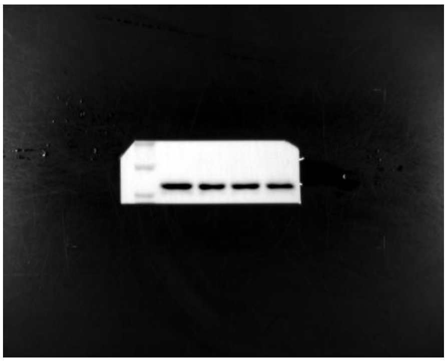

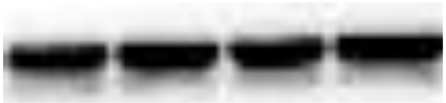

GAPDH

Figure 6

A

COL2A1 (right)

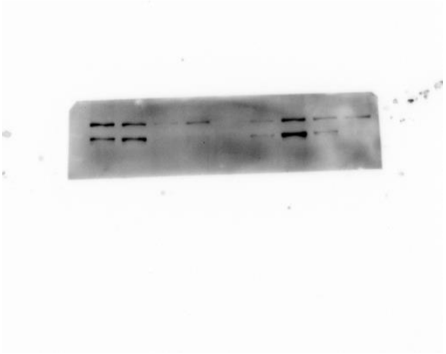

ACAN (left)

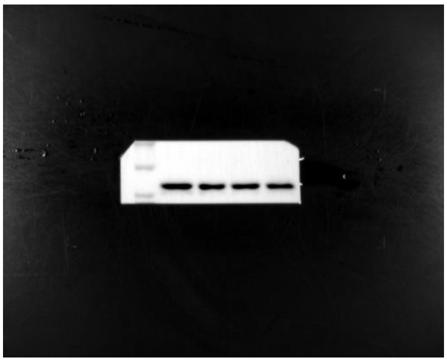

GAPDH
